# Complete Genome Sequence of *Campylobacter fetus* subsp. *testudinum* Strain 03-427^T^

**DOI:** 10.1128/genomeA.01002-13

**Published:** 2013-12-12

**Authors:** Maarten J. Gilbert, William G. Miller, Emma Yee, Martin J. Blaser, Jaap A. Wagenaar, Birgitta Duim

**Affiliations:** Department of Infectious Diseases and Immunology, Faculty of Veterinary Medicine, Utrecht University, Utrecht, The Netherlandsa; Produce Safety and Microbiology Research Unit, Agricultural Research Service, U.S. Department of Agriculture, Albany, California, USAb; Department of Medicine, New York University School of Medicine, New York, New York, USAc; Central Veterinary Institute of Wageningen UR, Lelystad, The Netherlandsd; WHO Collaborating Center for Campylobacter/OIE Reference Laboratory for Campylobacteriosis, Lelystad, The Netherlandse

## Abstract

*Campylobacter fetus* subsp. *testudinum* has been isolated from reptiles and humans. This *Campylobacter* subspecies is genetically distinct from other *C. fetus* subspecies. Here, we present the first whole-genome sequence for this *C. fetus* subspecies.

## GENOME ANNOUNCEMENT

*Campylobacter fetus* subsp. *testudinum* has been isolated from reptiles and humans and was recently described as a novel *C. fetus* subspecies (C. Fitzgerald, Z. C. Tu, M. Patrick, T. Stiles, A. J. Lawson, M. Santovenia, M. J. Gilbert, M. A. van Bergen, K. Joyce, J. Pruckler, S. Stroika, B. Duim, W. G. Miller, V. Loparev, J. C. Sinnige, P. I. Fields, R. V. Tauxe, M. J. Blaser, and J. A. Wagenaar, submitted for publication). *C. fetus* is an important animal pathogen and has been isolated from a diverse host range, including mammals, birds, and reptiles (1). *C. fetus* as isolated from reptiles (2, 3) is genetically distinct from mammal-associated *C. fetus* subsp. *fetus* and *C. fetus* subsp. *venerealis* (4), and it has been shown to cause infections in humans (5, 6). The genetic distance is larger between mammal- and reptile-associated *C. fetus* subspecies than within mammal-associated *C. fetus* subspecies. Here, we report the first whole-genome sequence of *C. fetus* subsp. *testudinum* strain 03-427^T^ (=LMG 27499^T^), which was isolated from a human (6).

Sequencing was performed using shotgun and paired-end reads obtained on a Roche 454 FLX genome sequencer. A total of 292,057 454 reads were assembled using the Newbler assembler (version 2.6) into a single scaffold of 22 contigs, which provided a draft genome sequence with a coverage of 62×. All 454 base calls were validated using 2,586,690 Illumina MiSeq reads, providing an additional 248× coverage. The scaffold gaps were filled as described previously (7). Assembly of the 03-427^T^ genome was validated using a bacterial optical restriction map (OpGen, Gaithersburg, MD). The sequences across the contig junctions and the S-layer (*sap*) locus were confirmed with Sanger sequencing. Homopolymeric G+C tracts were characterized using the high-depth MiSeq reads.

The circular genome size of *C. fetus* subsp. *testudinum* 03-427^T^ is 1,775,480 bp, with an average G+C content of 33.1%. No plasmids were identified. Protein-, rRNA- and tRNA-coding genes were identified as described previously (7). The genome was annotated based on that of *C. fetus* subsp. *fetus* strain 82-40 (accession no. NC_008599), with further annotation using Artemis (8) and the identification of Pfam domains (version 26.0 [9]). The genome contains 1,707 putative protein-coding genes (including 12 pseudogenes), 43 tRNA genes, and three rRNA operons. No obvious inserted or mobile elements were identified within the *C. fetus* subsp. *testudinum* 03-427^T^ genome, which contains 33 variable homopolymeric G+C tracts (≥8 bp). A CRISPR-Cas system and a type III restriction/modification system were identified. As in both *C. fetus* subsp. *fetus* and *C. fetus* subsp. *venerealis*, an S-layer coding region was present, as predicted by protein analyses and by hybridization (10–12). BLASTp analysis indicated a high degree of both synteny and similarity between the reptile- and mammal-associated *C. fetus* genomes; however, based on the core proteomes (i.e., proteins shared by all included strains), only 94% average amino acid identity was observed between the proteomes common to the reptile- and mammal-associated *C. fetus* subspecies, which is less than that between *C. fetus* subsp. *fetus* and *C. fetus* subsp. *venerealis* (>99%).

The whole-genome sequence of reptile-associated *C. fetus* provides a better understanding of the taxonomic structure within *C. fetus* and supports the proposal of *C. fetus* subsp. *testudinum*. Further genome analysis and comparison can provide valuable insights into the host adaptation, evolution, virulence, and the taxonomic structure of *C. fetus*.

### Nucleotide sequence accession number.

The complete genome sequence of *C. fetus* subsp. *testudinum* 03-427^T^ has been deposited in GenBank under the accession no. CP006833.
